# Indoor Air Quality: Lemon-Fresh Ozone

**Published:** 2007-07

**Authors:** Adrian Burton

Ionization air purifiers may be making our homes and offices more unhealthy places when cleaning products leave the air smelling lemon-fresh, according to research in the 1 April 2007 issue of *Environmental Science & Technology*. Scientists at the University of California, Irvine, report that ozone emitted by these purifiers reacts with certain volatile organic compounds such as limonene, producing potentially harmful levels of particulate matter (PM).

“In earlier work we showed ionization air purifiers, which are meant to remove particles from the air, to be producers of ozone, which itself causes a range of breathing problems and perhaps reduced resistance to infections,” says Sergey Nizkorodov, an assistant professor of chemistry at Irvine. “Now we show this ozone reacts with limonene entering the air from cleaning products to produce more PM than these machines can actually remove.” Limonene is used to scent cleaning products.

The researchers placed an ionization air purifier in an office equipped with a standard air exchange system. An ozone generator—a type of purifier that releases ozone to oxidize and theoretically neutralize volatile pollutants—was used as a comparison. d-limonene was injected into the room periodically to achieve concentrations similar to those encountered in offices after cleaning.

The ionization air purifier initially increased the ozone concentration by 5–15 ppb from a background level of 5 ppb, while the ozone generator raised it by some 250 ppb, according to Nizkorodov. With either machine operating, the limonene injections were accompanied by a spike of one to two orders of magnitude in the air’s PM_2.5_ and PM_0.1_ content—from 10^3^ to 10^5^ particles per cubic centimeter—that decayed over the next hour. “This means that in [limonene’s] presence, these air purifiers are actually air contaminators,” says Nizkorodov.

The researchers also produced a kinetic model to predict the net amount of PM produced by different machines under different indoor scenarios. “The results obtained with the model fitted the experimental data very well,” says Nizkorodov.

“The ultrafine particles produced by the reaction between volatile organic compounds and emitted ozone from ionization indoor air purifiers are of considerable interest since they are known to trigger oxidative stress and inflammation in the lungs,” says Claire Infante-Rivard, a professor of epidemiology at McGill University in Montréal. “The results of the present study should attract the attention of public health authorities regarding the safety of these devices.”

Peggy Jenkins, manager of the Indoor Exposure Assessment Branch at the California Air Resources Board, says that agency is currently developing a regulation to limit ozone emissions from indoor air-cleaning devices, especially ozone generators. “The Irvine results further support the need for this regulation,” she says. “In addition to reducing the directly emitted ozone and its associated impacts, the regulation will reduce the likelihood of particle and formaldehyde formation when limonene or other terpenes are present indoors.”

